# CT-derived extracellular volume and liver volumetry can predict posthepatectomy liver failure in hepatocellular carcinoma

**DOI:** 10.1186/s13244-023-01496-5

**Published:** 2023-09-12

**Authors:** Yangling Peng, Hao Tang, Yuanying Huang, Xiaoqian Yuan, Xing Wang, Zijuan Ran, Wei Deng, Renwei Liu, Xiaosong Lan, Hesong Shen, Jiuquan Zhang

**Affiliations:** 1 Department of Radiology, Chongqing University Cancer Hospital & Chongqing Cancer Institute & Chongqing Cancer Hospital, Chongqing, 400030 People’s Republic of China; 2 Department of Hematology, Chongqing General Hospital, University of the Chinese Academy of Sciences, Chongqing, People’s Republic of China

**Keywords:** Posthepatectomy liver failure, Hepatocellular carcinoma, Extracellular space, Computed tomography

## Abstract

**Objectives:**

Posthepatectomy liver failure (PHLF) is a severe complication of liver resection. We aimed to develop and validate a model based on extracellular volume (ECV) and liver volumetry derived from computed tomography (CT) for preoperative predicting PHLF in resectable hepatocellular carcinoma (HCC) patients.

**Methods:**

A total of 393 resectable HCC patients from two hospitals were enrolled and underwent multiphasic contrast-enhanced CT before surgery. A total of 281 patients from our hospital were randomly divided into a training cohort (*n* = 181) and an internal validation cohort (*n* = 100), and 112 patients from another hospital formed the external validation cohort. CT-derived ECV was measured on nonenhanced and equilibrium phase images, and liver volumetry was measured on portal phase images. The model is composed of independent predictors of PHLF. The under the receiver operator characteristic curve (AUC) and calibration curve were used to reflect the predictive performance and calibration of the model. Comparison of AUCs used the DeLong test.

**Results:**

CT-derived ECV, measured future liver remnant (mFLR) ratio, and serum albumin were independent predictors for PHLF in resectable HCC patients. The AUC of the model was significantly higher than that of the ALBI score in the training cohort, internal validation cohort, and external validation cohort (all *p* < 0.001). The calibration curve of the model showed good consistency in the training cohort and the internal and external validation cohorts.

**Conclusions:**

The novel model contributes to the preoperative prediction of PHLF in resectable HCC patients.

**Critical relevance statement:**

The novel model combined CT–derived extracellular volume, measured future liver remnant ratio, and serum albumin outperforms the albumin–bilirubin score for predicting posthepatectomy liver failure in patients with resectable hepatocellular carcinoma.

**Key points:**

• CT-derived ECV correlated well with the fibrosis stage of the background liver.

• CT-derived ECV and mFLR ratio were independent predictors for PHLF in HCC.

• The AUC of the model was higher than the CT-derived ECV and mFLR ratio.

• The model showed a superior predictive performance than that of the ALBI score.

**Graphical Abstract:**

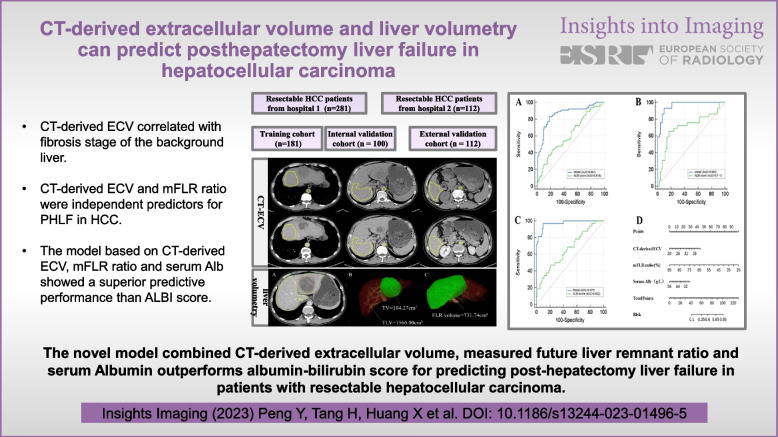

**Supplementary Information:**

The online version contains supplementary material available at 10.1186/s13244-023-01496-5.

## Introduction

Surgical resection is the most effective treatment method for patients with hepatocellular carcinoma (HCC) [1]. Posthepatectomy liver failure (PHLF) is a severe complication of liver resection, and among these complications, PHLF directly leads to almost half of the deaths [2–4]. The reported incidences of PHLF have ranged from 0.7 to 39.6% [3, 5]. The occurrence of PHLF depends on the quality and quantity of the future remnant liver [6, 7]. Therefore, it is crucial to accurately preoperatively evaluate the function and volume of future residual liver to prevent the occurrence of PHLF.

Zou et al. [8] found that the albumin–bilirubin (ALBI) score has been proven to be a reliable clinical model for predicting PHLF in HCC patients. This clinical model only includes serum bilirubin and albumin and eliminates the influence of subjective parameters such as encephalopathy and ascites. However, the ALBI score reflects the function of the whole liver and not just the future remnant liver.

Multiphasic contrast-enhanced computed tomography (CT) is a widely recommended modality for the preoperative assessment of HCC [9, 10] and is a routine clinical examination. The histopathology feature of liver fibrosis is the expansion of extracellular volume (ECV) caused by the deposition of collagen and matrix proteins [11]. Patients with liver fibrosis or cirrhosis have an impaired hepatic regenerative capacity [6]. A previous study [12] found that hepatic insufficiency was closely related to the severity of liver fibrosis. Previous studies [4, 11, 13–15] have shown that ECV can predict the degree of liver fibrosis. Our previous study [16] found that CT-derived ECV can predict PHLF in resectable HCC patients. However, in our previous study, the nomogram based on CT-derived ECV only considered the function of the future remnant liver and did not incorporate the volume of the future remnant liver. Assessment of the future liver remnant (FLR) volume by CT has been shown to be correlated with clinical outcomes in HCC patients [17, 18].

Our hypothesis is that the model combining CT-derived ECV and liver volumetry provides a more precise and more individualized tool to predict PHLF than CT-derived ECV alone. In this study, we aimed to develop and validate a model based on CT-derived ECV and FLR for predicting PHLF in resectable HCC patients and to compare the preoperative prediction efficacy of the model with the ALBI score.

## Materials and methods

### Patients

This retrospective study was approved by the Institutional Review Board of the two participating hospitals. Informed consent was waived. The inclusion criteria were as follows: (1) histopathologically confirmed HCC after hepatectomy, (2) preoperative liver function Child–Pugh stage (A/B), and (3) multiphasic contrast-enhanced CT examination and serum marker testing completed within 2 weeks before the operation. The exclusion criteria were as follows: (1) patients with other tumors and (2) patients who had experienced pre-operative portal vein embolization/ablation/chemoembolization/radioembolization or chemotherapy.

A total of 281 consecutive patients (between June 2013 and June 2022, at Chongqing University Cancer Hospital [CQUCH]) who met the inclusion criteria were enrolled and then randomly divided into a training cohort (*n* = 181) and an internal validation cohort (*n* = 100). A total of 112 consecutive patients (between November 2019 and July 2022, at Chongqing General Hospital [CQGH]) who met the same inclusion criteria were enrolled and formed the external validation cohort. Some of these patients (121 patients in CQUCH and 81 patients in CQGH) were analyzed as a subcohort of another published study [16].

### Collection of data

Preoperative laboratory data, comorbidities, surgical data, and histopathological data were recorded (detailed in supplementary materials). The ALBI score was calculated using the following formula [8]: ALBI score = 0.66 × log10 (Tbil [μmol/L]) − 0.085 × (Alb [g/L]). Consensus definition and severity grading of PHLF by the International Study Group of Liver Surgery (ISGLS) [7].

### CT protocol

Multiphasic contrast-enhanced CT was conducted for all patients using two CT scanners (SOMATOM Definition AS and SOMATOM DRIVE, Siemens Healthineers). The scanning range was from the lung base to the iliac crest. An iodinated contrast agent (Ioversol, 320 mg/mL iodine, HENGRUI Medicine) was injected intravenously at a rate of 3.0–3.5 mL/s for a total of 80–100 mL (1.5 mL/kg of body weight). Nonenhanced images were acquired using the conventional helical scan mode. Bolus tracking was used. Arterial phase scanning began 7 s after the trigger attenuation threshold (100 HU) reached the level of the supraceliac abdominal aorta. Portal phase scanning began at a delay of 30 s after the arterial phase scanning, and the equilibrium phase (EP) began at a delay of 90 s after the portal phase scanning. The scanning parameters were as follows: tube voltage = 120 kV, reference tube current = 180 mAs, increment collimation = 128 × 0.6 mm, pitch = 1.0, and rotation time = 0.5 s. The reconstruction parameters were as follows: thickness = 1.5 mm, increment = 1 mm, and soft-tissue convolution kernel (I31). CT images were reconstructed with the projection-based material decomposition software using a standard reconstruction kernel.

Extracellular volume analysis is detailed in supplementary materials.

### Liver volumetry analysis

With the known clinical and surgery data, preoperative total liver volume (TLV), tumor volume (TV), and future liver remnant (FLR) volume were measured by radiologist 1 (15 years of experience in abdominal CT imaging) on the portal phase images (thickness = 1.5 mm) using a postprocessing workstation (syngo.via VB20A, Dual Energy, Siemens Healthineers, Forchheim, Germany). These measurements included the volumes of the intrahepatic blood vessels and bile ducts to ensure uniformity and reproducibility of the liver. The CT liver volumetry analysis is shown in Additional file 1: Fig. S1. The measured FLR (mFLR) ratio was calculated using the following formula:

$$\mathrm{mFLR}\;\mathrm{ratio }\left(\mathrm{\%}\right) = \frac{\mathrm{FLR\;volume}}{(\mathrm{TLV }-\mathrm{ TV})} \times 100$$

### Measurement reliability

To assess the intraobserver and interobserver reliability of the CT-derived ECV and CT liver volumetry (TLV, TV, FLR volume), radiologist 1 and radiologist 2 (10 years of experience in abdominal CT imaging) remeasured the values 4 at weeks after the first assessment using the same technique at the first measurement.

### Statistical analysis

All data analyses were performed using SPSS statistics version 25.0 (IBM). The Kolmogorov–Smirnov test was used to assess the normality of the quantitative data distribution. Continuous variables are expressed as the mean ± standard deviation (SD) or median and interquartile range, whereas categorical variables are presented as numbers and percentages. The intraclass correlation coefficients (ICCs) were used to assess the intraobserver and interobserver reliability. The association between CT-derived ECV and the fibrosis stage of the background liver was analyzed by using Spearman’s rank correlation. For the univariate analysis, comparisons between the patients with/without PHLF were performed using the Wilcoxon rank sum test or Student’s *t* test for continuous variables and the *χ*^2^ test or Fisher’s exact test for categorical variables. Statistically significant preoperative variables in the univariate analysis (*p* value < 0.05) were included in the multivariate logistic regression analysis. The *β* coefficients from the multivariate logistic regression analysis results were used to form the nomogram to assess the risk for PHLF using the R software (version 3.6.2). The predictive performance of the model and ALBI score was assessed by the areas under the receiver operator characteristic curve (AUCs). The DeLong test was used to compare the AUCs. The calibration curve was used to reflect the calibration of the model. A *p* value < 0.05 (two-tailed) was considered statistically significant.

## Results

### Patient characteristics

The characteristics of the study patients are summarized in Tables 1 and 2. In our study, 281 resectable HCC patients from CQUCH who met the inclusion criteria were enrolled and randomly assigned to a training cohort (*n* = 181) and an internal validation cohort (*n* = 100). A total of 112 consecutive patients from CQGH who met the inclusion criteria were included in an external validation cohort. Among all the cohorts, PHLF occurred in 126 patients (32.06%). The incidence of PHLF was 35.91% (65/181) in the training cohort, 29.00% (29/100) in the internal validation cohort, and 28.57% (32/112) in the external validation cohort. The mean ages in the training cohort, internal validation cohort, and external validation cohort were 53.68 years ± 11.30 (range, 24–79), 55.30 years ± 11.23 (range, 13–79), and 54.29 years ± 12.93 (range, 13–79), respectively.

**Table 1:** The clinical and pathological data of the study patients

Variable	Training cohort (*n* = 181)			Internal validation cohort (*n* = 100)			External validation cohort (*n* = 112)		
	Without PHLF (*n* = 116)	With PHLF (*n* = 65)	*p* value	Without PHLF (*n* = 71)	With PHLF (*n* = 29)	*p* value	Without PHLF (*n* = 80)	With PHLF (*n* = 32)	*p* value
Age (years)	54.81 ± 11.44	51.66 ± 10.83	0.072	55.77 ± 11.88	54.14 ± 9.52	0.511	54.38 ± 12.91	54.09 ± 13.20	0.918
Male (*N*)	94 (81.03%)	56 (86.15%)	0.380	65 (91.55%)	25 (86.21%)	0.419	61 (76.25%)	28 (87.50%)	0.183
PLT count (10^9^/L)	164.50 (129.00, 205.25)	167.00 (131.50, 196.50)	0.920	147 (101, 193)	143 (93, 179)	0.352	179.50 (145.50, 212.50)	173.00 (137.00, 198.00)	0.704
Serum ALT (μmol/L)	36.00 (25.00, 53.00)	44.00 (28.65, 65.00)	0.065	32 (24, 42)	31 (27, 47)	0.521	35.00 (26.00, 47.00)	42.50 (28.88, 74.00)	0.091
Serum AST (μmol/L)	35.00 (26.00, 50.00)	44.00 (28.00, 61.70)	0.059	31 (24, 51)	34 (26, 50)	0.427	37.00 (26.00, 57.03)	43.50 (26.05, 61.85)	0.664
Serum Alb (g/L)	40.49 ± 5.35	38.69 ± 5.47	0.033*	40.07 ± 4.91	37.86 ± 4.75	0.042	42.25 ± 4.49	40.61 ± 4.90	0.091
Serum Tbil (μmol/L)	13.96 (11.40, 18.51)	15.57 (10.73, 29.69)	0.198	14.00 (9.15, 19.75)	20.71 (16.19, 30.80)	< 0.001	13.22 (10.35, 15.16)	15.81 (11.88, 28.99)	0.001
Serum Cr (μmol/L)	67.00 (55.63, 75.80)	65.20 (54.60, 72.35)	0.304	68.00 (59.60, 80.00)	70.50 (56.00, 85.15)	0.958	63.25 (51.66, 72.24)	65.30 (52.35, 71.37)	0.995
Serum Hct (L/L)	0.41 (0.36, 0.45)	0.41 (0.37, 0.44)	0.727	0.38 (0.30, 0.43)	0.43 (0.36, 0.44)	0.079	0.40 (0.32, 0.43)	0.41 (0.38, 0.43)	0.177
Serum INR	0.98 (0.92, 1.07)	0.97 (0.91, 1.05)	0.374	1.02 (0.97, 1.08)	0.99 (0.95, 1.06)	0.175	1.08 (1.02, 1.13)	1.10 (1.02, 1.16)	0.713
Serum PT (s)	11.60 (10.90, 12.58)	11.40 (10.65, 12.10)	0.067	12.00 (11.50, 13.00)	11.70 (11.30, 12.05)	0.080	11.60 (10.90, 12.67)	11.35 (10.55, 11.98)	0.069
Serum PTA (%)	92.38 ± 18.24	96.72 ± 14.60	0.102	86.69 ± 13.44	88.90 ± 12.83	0.451	91.37 ± 16.75	97.78 ± 16.90	0.071
Operation time, surgery (min)	357.00 (266.25, 504.25)	425 (270, 529)	0.288	392 (285, 497)	350 (228, 450)	0.220	297.50 (210.75, 450.00)	427.50 (281.25, 599.00)	0.013
Hepatic inflow occlusion duration	0 (0, 50)	30 (15, 66)	< 0.001*	0 (0, 0)	45.00 (15.00, 75.00)	< 0.001	0 (0, 23.75)	15.00 (0, 44.25)	0.024
Blood loss, surgery (mL)	200 (100, 400)	300 (110, 500)	0.164	200 (100, 400)	200 (100, 250)	0.152	200 (100, 475)	300 (123, 500)	0.134
Blood transfusion, surgery (mL)	0 (0, 0)	0 (0, 0)	0.495	0 (0, 0)	0 (0, 0)	0.663	0 (0, 0)	0 (0, 0)	0.764
Hepatectomy, surgery			< 0.001*			0.014			0.153
Minor hepatectomy	101 (87.07%)	34 (52.31%)		60 (84.51%)	18 (62.07%)		67 (83.75%)	23 (71.88%)	
Major hepatectomy	15 (12.93%)	31 (47.69%)		11 (15.49%)	11 (37.93%)		13 (16.25%)	9 (28.12%)	
Hypertension (*N*)	23 (19.83%)	9 (13.85%)	0.312	8 (11.27%)	4 (13.79%)	0.724	11 (13.75%)	3 (9.38%)	0.754
Diabetes mellitus (*N*)	13 (11.21%)	3 (4.62%)	0.134	5 (7.04%)	0	0.337	9 (11.25%)	2 (6.25%)	0.651
The presence of viral hepatitis (*N*)	85 (73.28%)	38 (58.46%)	0.060	51 (71.83%)	22 (75.86%)	0.680	58 (72.50%)	20 (62.50%)	0.298
Maximum diameter, pathology (cm)	4.00 (2.35, 7.00)	4.50 (2.50, 8.90)	0.344	4.50 (2.50, 8.00)	5.30 (2.50, 6.50)	0.933	4.75 (2.35, 8.00)	3.25 (2.28, 6.45)	0.242
Fibrosis stage, pathology			0.022*			0.009			0.009
S0	42 (36.21%)	16 (24.62%)		31 (43.66%)	3 (10.34%)		41 (51.25%)	5 (15.63%)	
S1	17 (14.66%)	11 (16.92%)		4 (5.63%)	1 (3.45%)		11 (13.75%)	6 (18.75%)	
S2	27 (23.28%)	7 (10.77%)		7 (9.86%)	3 (10.34%)		10 (12.50%)	5 (15.63%)	
S3	13 (11.21%)	15 (23.08%)		10 (14.08%)	6 (20.69%)		10 (12.50%)	8 (25.00%)	
S4	17 (14.66%)	16 (24.62%)		19 (26.76%)	16 (55.17%)		8 (10.00%)	8 25.00%)	
Steatosis, pathology (*N*)	8 (6.90%)	4 (6.15%)	0.847	4 (5.63%)	0	0.458	8 (10.00%)	3 (9.38%)	0.920
Tumor thrombus, pathology (*N*)	30 (25.86%)	25 (38.46%)	0.077	19 (26.76%)	9 (31.03%)	0.666	21 (26.25%)	9 (28.13%)	0.840
Clinical models									
Child − Pugh stage			0.559			0.139			0.791
A	102 (87.93%)	59 (90.77%)		63 (88.73%)	29 (100%)		76 (95.00%)	30 (93.75%)	
B	14 (12.07%)	6 (9.23%)		8 (11.27%)	0		4 (5.00%)	2 (6.25%)	
MELD score	15.89 ± 4.57	15.39 ± 4.56	0.476	16.40 ± 4.46	17.78 ± 2.98	0.132	15.73 ± 4.15	17.44 ± 5.03	0.066
ALBI score	− 2.67 (− 2.98, − 2.40)	− 2.56 (− 2.82, − 1.99)	0.010*	− 2.62 (− 2.94, − 2.36)	− 2.17 (− 2.67, − 2.05)	0.001	− 2.86 (− 3.11, − 2.61)	− 2.67 (− 2.95, − 2.28)	0.008

*Note*: Data are presented as *n* (percentage), median (interquartile range), or mean ± standard deviation

*Abbreviations*: *PHLF* Posthepatectomy liver failure, *PLT counts* Platelet counts, *ALT* Alanine aminotransferase, *AST* Aspartate aminotransferase, *Alb* Albumin, *Cr* Creatinine, *Tbil* Total bilirubin, *Hct* Hematocrit, *INR*, International normalized ratio, *PT* Prothrombin time, *PTA* Prothrombin activity, *MELD* Model for end-stage liver disease, *ALBI* Albumin–bilirubin

^*^The comparison between patients without PHLF and patients with PHLF in the training cohort. *p* value < 0.05 means significant

**Table 2:** Computed tomography data of the study patients

Variable	Training cohort (*n* = 181)			Internal validation cohort (*n* = 100)			External validation cohort (*n* = 112)		
	Without PHLF (*n* = 116)	With PHLF (*n* = 65)	*p* value	Without PHLF (*n* = 71)	With PHLF (*n* = 29)	*p* value	Without PHLF (*n* = 80)	With PHLF (*n* = 32)	*p* value
Liver cirrhosis (*N*)	50 (43.10%)	23 (35.38%)	0.310	22 (30.99%)	7 (24.14%)	0.493	34 (42.50%)	12 (37.50%)	0.627
Ascites (*N*)	11 (9.48%)	5 (7.69%)	0.684	8 (11.27%)	1 (3.45%)	0.393	8 (10.00%)	4 (12.50%)	0.961
CT-derived ECV	25.83 (23.26, 28.08)	29.78 (26.41, 32.94)	< 0.001*	25.64 (23.25, 30.15)	31.03 (27.67, 34.93)	< 0.001	26.03 (22.94, 28.11)	28.88 (27.46, 32.40)	< 0.001
TV (cm^3^)	43.85 (12.85, 136.75)	67.20 (22.75, 419.00)	0.035*	35.00 (8.12, 226.00)	71.30 (24.25, 229.80)	0.676	37.83 (5.41, 247.28)	12.11 (3.96, 89.87)	0.130
TLV (cm^3^)	1262.00 (1046.75, 1521.00)	1348.00 (1205.50, 1574.00)	0.579	1242.88 (1025.56, 1508.00)	1303.00 (1112.33, 1535.00)	0.261	1401.00 (1171.00, 1571.00)	1411.50 (1255.75, 1586.25)	0.495
FLR volume (cm^3^)	924.42 ± 219.33	794.71 ± 212.56	< 0.001*	874.40 ± 195.61	748.54 ± 213.63	0.005	956.98 ± 212.99	866.66 ± 151.06	0.031
mFLR ratio (%)	81.36 (78.26, 84.63)	66.27 (57.22, 79.85)	< 0.001*	81.93 (79.52, 84.43)	61.22 (53.71, 66.05)	< 0.001	79.89 (76.90, 83.11)	67.45 (61.03, 70.84)	< 0.001

*Note*: Data are presented as *n* (percentage), median (interquartile range), or mean ± standard deviation

*Abbreviations*: *PHLF* Posthepatectomy liver failure, *CT* Computed tomography, *ECV* Extracellular volume, *TLV* Total liver volume, *TV* Tumor volume, *FLR* Future liver remnant, *mFLR* Measured FLR

^*^The comparison between patients without PHLF and patients with PHLF in the training cohort. *p* value < 0.05 means significant

Spearman correlation analysis revealed that CT-derived ECV had a strong correlation with the postoperative pathological fibrosis stage of the background liver in the training cohort (*p* < 0.001, *r* = 0.701; Additional file 1: Fig. S2).

### The intraobserver and interobserver reliability of CT-derived ECV and CT volumetry

The intraobserver and interobserver reliability of CT-derived ECV and CT volumetry (TLV, TV, FLR volume) were good in the training, internal validation, and external validation cohorts, and all the ICC values were greater than 0.80 (Additional file 1: Table S1). Therefore, the average value of the two radiologist measurements was used for further analysis.

### Independent predictors of posthepatectomy liver failure in the training cohort

The univariate analyses for PHLF in the training cohort are shown in Tables 1 and 2. CT-derived ECV (29.78 vs. 25.83; *p* < 0.001) and TV (67.20 cm^3^ vs. 43.85 cm^3^; *p* = 0.035) were higher in patients with PHLF than in patients without PHLF. The FLR volume (794.710 cm^3^ ± 212.562 vs. 924.420 cm^3^ ± 219.326; *p* < 0.001), mFLR ratio (66.27% vs. 81.36%; *p* < 0.001), and serum Alb (38.69 g/L ± 5.473 vs. 40.49 g/L ± 5.348; *p* = 0.033) were lower in patients with PHLF than in patients without PHLF. Hepatectomy (*p* < 0.001) and fibrosis stage (*p* = 0.022) were significantly associated with the development of PHLF.

These seven preoperative risk factors were included in the multivariate logistic regression analysis. After a method of input, three factors were selected. CT-derived ECV (odds ratio [OR]: 1.214, 95% confidence interval [CI]: 1.067–1.381, *p* = 0.003), mFLR ratio (OR: 0.897, 95% CI: 0.852–0.944, *p* < 0.001), and serum Alb (OR: 0.923, 95% CI: 0.855–0.997, *p* = 0.043) were independent predictors for PHLF in patients with resectable HCC (Table 3).

**Table 3:** Multivariable logistic regression analysis for PHLF prediction in resectable HCC patients

Variable	*B*	BE	Walds	*P*	Exp(*B*)	95% CI
CT-derived ECV	0.194	0.066	8.706	0.003	1.214	1.067–1.381
mFLR ratio (%)	− 0.109	0.026	17.389	< 0.001	0.897	0.852–0.944
Serum Alb (g/L)	− 0.080	0.039	4.109	0.043	0.923	0.855–0.997
Constant	5.924	3.279	3.264	0.071	374.029	

*Note*: *β* is the regression coefficient. *p* value < 0.05 means significant

*Abbreviations*: *OR* Odds ratio, *CI* Confidence interval, *CT* Computed tomography, *ECV* Extracellular volume, *mFLR* Measured future liver remnant, *Alb* Albumin, *PHLF* Posthepatectomy liver failure, *HCC* Hepatocellular carcinoma

The median ALBI score of the patients with PHLF was significantly higher than that of the patients without PHLF (− 2.56 vs. − 2.67; *p* = 0.010). No significant differences in the Child–Pugh stage or MELD score between the patients with and without PHLF were found (both *p* > 0.05).

### Development of the model

**Fig. 1:**
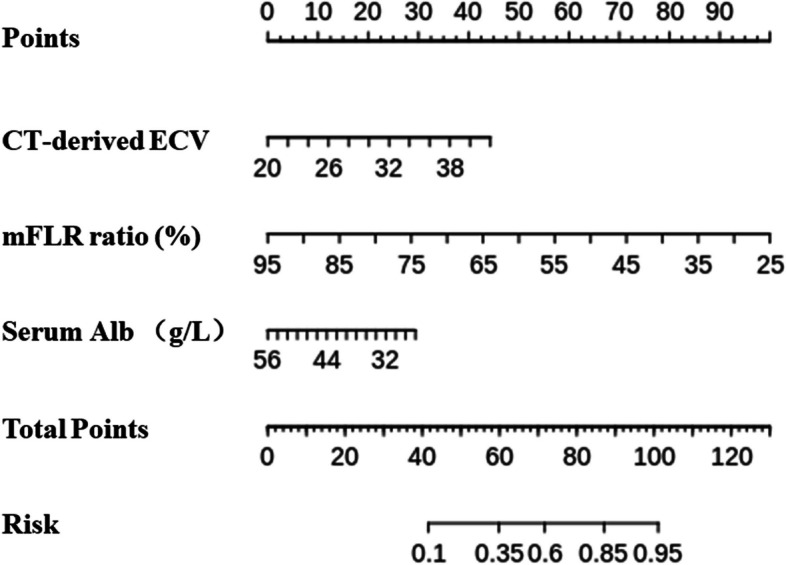
The nomogram was a visualization of the model for predicting PHLF in patients with resectable HCC. The model was combined by CT-derived ECV, mFLR ratio, and serum Alb. PHLF, posthepatectomy liver failure; HCC, hepatocellular carcinoma; CT, computed tomography; ECV, extracellular volume; mFLR, measured future liver remnant; Alb, albumin

**Fig. 2:**
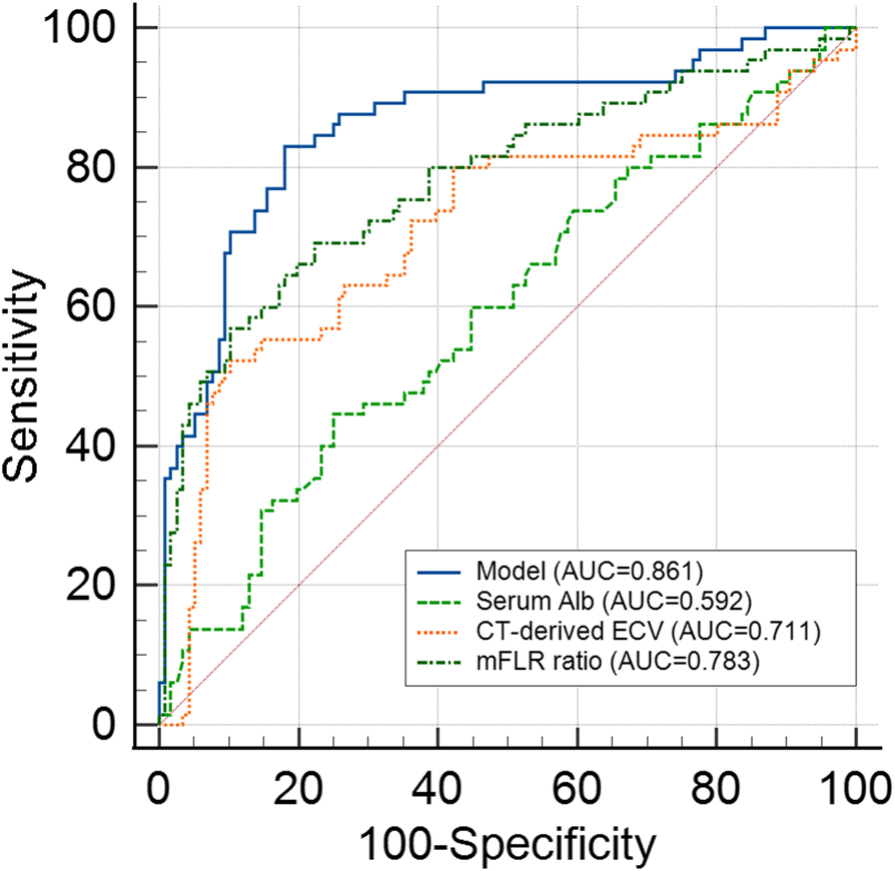
Receiver operating characteristic (ROC) curve analyses of the model, CT-derived ECV, mFLR ratio, serum Alb, and hepatic inflow occlusion duration to predict PHLF. The model for PHLF prediction in the training cohort had a significantly higher AUC (0.861, 95% CI: 0.802–0.908) than those of CT-derived ECV, mFLR ratio, and serum Alb (DeLong test: all *p* < 0.001). PHLF, posthepatectomy liver failure; HCC, hepatocellular carcinoma; CT, computed tomography; ECV, extracellular volume; mFLR, measured future liver remnant; Alb, albumin; AUC, area under the curve

**Fig. 3:**
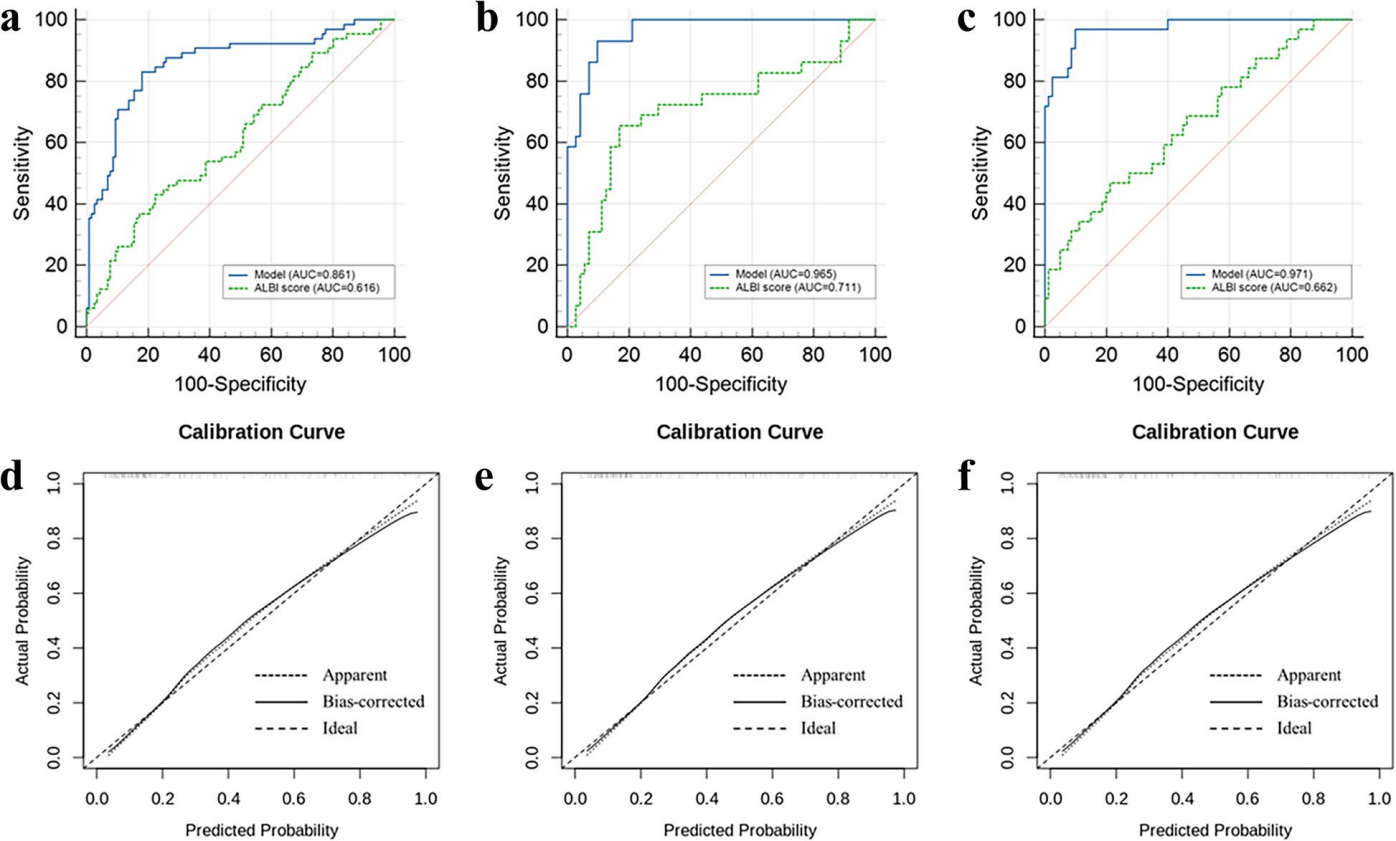
Comparison of predictive efficacy for PHLF between the model and the ALBI score. The AUC of the model was significantly superior to that of the ALBI score in the training cohort (**a**), internal validation cohort (**b**), and external validation cohort (**c**) (DeLong test: *p* < 0.001, *p* < 0.001, and *p* < 0.001, respectively). The calibration curves of the model in the training cohort (**d**), internal validation cohort (**e**), and external validation cohort (**f**) showed good consistency. PHLF, posthepatectomy liver failure; ALBI, albumin–bilirubin; AUC, area under the curve

Based on these significant independent predictors, we constructed a model for PHLF prediction. The nomogram was visualization of the model (Fig. 1). The model for PHLF prediction in the training cohort had a significantly higher AUC (0.861, 95% CI: 0.802–0.908) than those of the CT-derived ECV (0.711, 95% CI: 0.639–0.776, *p* < 0.001), mFLR ratio (0.783, 95% CI: 0.716–0.841, *p* = 0.009), and serum Alb (0.592, 95% CI: 0.516–0.664, *p* < 0.001) (Fig. 2). The model was well calibrated according to the calibration curve (Fig. 3D).

### Comparison of the predictive efficacy for PHLF between the model and the ALBI score in the training cohort

There was no significant difference in the Child–Pugh stage or MELD score between the patients with and without PHLF (both *p* > 0.05). The AUC of the model was significantly higher than that of the ALBI score (0.861, 95% CI, 0.802–0.908 vs. 0.616, 95% CI, 0.541–0.687; *p* < 0.001) (Fig. 3A, Table 4). The optimal cutoff value of the model for predicting PHLF in the training cohort was 0.325, with a sensitivity of 83.10% and a specificity of 81.90% for predicting PHLF (Table 4).

**Table 4:** Comparison of predictive efficacy for PHLF between the model and ALBI score

	**Training cohort**		**Internal validation cohort**		**External validation cohort**	
	**Model**	**ALBI score**	**Model**	**ALBI score**	**Model**	**ALBI score**
**AUC (95% CI)**	0.861 (0.802–0.908)	0.616 (0.541–0.687)	0.965 (0.907–0.991)	0.711 (0.611–0.797)	0.971 (0.921–0.994)	0.662 (0.566 − 0.748)
**Youden index**	0.650	0.207	0.832	0.486	0.869	0.256
**Cutoff value**	0.325	− 2.383	0.232	− 2.257	0.179	− 2.606
**Sensitivity**	0.831	0.431	0.931	0.655	0.969	0.469
**Specificity**	0.819	0.776	0.901	0.831	0.900	0.788
***p*** **value**^**a**^	< 0.001		< 0.001		< 0.001	

*Note*: Cutoff values were chosen to achieve the Youden index. *p* value < 0.05 means significant

*Abbreviations*: *AUC* Area under the receiver operating characteristic, *CI* Confidence interval, *ALBI* Albumin–bilirubin, *PHLF* Posthepatectomy liver failure

^a^The significance of the differences between the model and the ALBI score was assessed by the DeLong test

### Internal and external validation of the model

The background characteristics of the patients in the internal and external validation cohorts are also shown in Tables 1 and 2. In the internal and external validation cohorts, the AUC of the model was significantly higher than that of the ALBI score (0.965, 95% CI: 0.907–0.991 vs. 0.711, 95% CI: 0.611–0.797, *p* < 0.001; 0.971, 95% CI: 0.921–0.994 vs. 0.662, 95% CI: 0.566–0.748, *p* < 0.001, respectively) (Fig. 3B, C, Table 4). According to the calibration curve, the model showed good agreement between the prediction and actual observation in the internal and external validation cohorts (Fig. 3E, F). The optimal cutoff values of the model for predicting PHLF in the internal validation and external validation cohorts were 0.232 and 0.179, respectively, and their sensitivity and specificity for predicting PHLF are shown in Table 4.

## Discussion

In the present study, we developed and validated a model for PHLF prediction in patients with resectable HCC. The model incorporated three preoperative independent factors, including CT-derived ECV, mFLR ratio, and serum Alb. The model displayed a good predictive performance for the preoperative prediction of PHLF and had a good agreement between the probability and actual observations in the training cohort and the internal and external validation cohorts. In addition, the predictive performance of the model was significantly superior to that of the ALBI score in the training cohort (0.861 vs. 0.616), internal validation cohort (0.965 vs. 0.711), and external validation cohort (0.971 vs. 0.662). The model may be helpful for providing pretreatment consultation for patients who are suitable for hepatectomy.

The present study and our previous study [16] have both confirmed that CT-derived ECV of the background liver is strongly correlated with the postoperative pathological fibrosis stage, and CT-derived ECV is an independent predictor of PHLF. A higher ECV means more severe liver fibrosis and a higher risk for PHLF in patients with resectable HCC. Our results are consistent with those of previous studies [11, 13, 14, 19]. The mFLR ratio was identified as a significant independent predictor of PHLF in resectable HCC patients. Patients with a smaller mFLR ratio are at a higher risk for PHLF, which is consistent with previous studies [18, 20]. The AUC of the mFLR ratio for PHLF prediction was 0.783 in the present study, which was near the AUC of 0.753 in a previous study [5]. Serum Alb has been confirmed to be associated with PHLF [21, 22], and our results are consistent with those of previous studies. The lower the serum Alb is, the higher the risk for PHLF. Hepatectomy (major or minor) was a significant factor of PHLF in the present study but was not included as a final independent predictor, which was consistent with a previous study [23].

The performance of our model for PHLF prediction was superior to that of the clinical models in the training cohort and the internal and external validation cohorts (0.861 vs. 0.616, *p* < 0.001; 0.965 vs. 0.711, *p* < 0.001; 0.971 vs. 0.662, *p* < 0.001, respectively). The model combining CT-derived ECV, mFLR ratio, and serum Alb can be used as a promising noninvasive method to evaluate the function and volume of future liver remnants. On the one hand, laboratory markers and clinical models (Child–Pugh stage, MELD score, and ALBI score) are usually used to assess hepatic functional reserve. These parameters can only evaluate overall liver function and cannot accurately evaluate regional liver function, especially in the presence of liver function heterogeneity [24, 25]. On the other hand, the mFLR ratio can only reflect the ratio of FLR volume to liver volume with normal liver function (TLV − TV). One shortcoming of volumetry is the fact that volumetric assessment of the future liver remnant does not consider the quality of the future remnant liver parenchyma, which might be impaired by underlying liver parenchymal diseases, such as fibrosis, cirrhosis, or steatosis. Therefore, the model combining the evaluation of the function and volume of the FLR may be a valuable method to predict PHLF in routine clinical practice; however, this does not add any additional examination to those tests that are already routinely performed.

This study still had several limitations. First, this study is a retrospective study based on two centers in Chongqing, China. To further verify the clinical reliability and effectiveness of the novel model in this study, a prospective large-sample (more centers and larger samples from different regions and countries) study is needed. Second, the CT-derived ECV and mFLR ratio in this study are both based on CT images, while Gd-EOB-DTPA-enhanced magnetic resonance imaging (MRI) can perform both volumetric and functional analyses [25–27]. The clinical effectiveness of the model based on CT needs to be compared with Gd-EOB-DTPA-enhanced MRI in the future. Third, the univariate analysis showed that hepatectomy (major or minor) was a significant factor of PHLF in this study, and most of the patients had minor hepatectomy (135/181). Thus, a subgroup analysis of different hepatectomies (major or minor) is needed in the future. However, in the subgroup analysis of this study, the results showed no statistical difference in the predictive performance of the model. Fourth, the patients with PHLF were not further classified in detail (grade A/B/C) in the present study. Due to the small number of positive cases, the data after grouping were very unbalanced, and most patients were grade A or B. Therefore, further subgroup analysis is needed.

## Conclusions

The novel model, combining CT-derived ECV, mFLR ratio, and serum Alb, showed a superior prediction of PHLF in patients with resectable HCC than the ALBI score. As a feasible and promising noninvasive tool, this model may provide a new strategy for the preoperative prediction of PHLF.

### Supplementary Information

**Additional file 1: Fig. S1. **Sample for CT liver volumetry analysis. A 59-year-old man with HCC, underwent a major hepatectomy (resection of left lobe of liver). In the preoperative baseline axial portal phase CT image(A), handcrafted ROIs were drawn along the margins of the tumor (TV, ROI contoured in orange), the total liver (TLV, ROI contoured in green), and the future liver remnant (FLR volume, ROI contoured in yellow). Volume-rendered image of TV (B) and FLR volume (C) in green, TLV in red, hepatic veins and portal veins in golden. *CT* Computed tomography, *ROI* Region of interest, *TV* Tumor volume, *TLV* Total liver volume, *FLR *Future liver remnant. **Fig. S2.** Boxplot showed a strong correlation between CT-derived ECV and the postoperative pathological fibrosis stage of the background liver (*p* < 0.001, *r* = 0.701). *CT* Computed tomography, *ECV* Extracellular volume. **Fig. S3.** Boxplot showed that there was a significant difference in the mean value of CT-derived ECV between subgroup S0-2 and S3-4 (25.34 ± 3.03 vs. 31.17 ± 4.40, *p* < 0.001). *CT* Computed tomography, *ECV* Extracellular volume. **Table S1.** Intraobserver and interobserver reliability of CT-derived ECV and CT liver volumetry. Abbreviations: *CI* Confidence interval, *CT* Computed tomography, *ECV *Extracellular volume, *TLV* Total liver volume, *TV* Tumor volume, *FLR* Future liver remnant.

## Data Availability

The datasets generated during and/or analyzed during the current study are available from the corresponding author upon reasonable request.

## Abbreviations

Alb: Albumin
ALBI: Albumin–bilirubin
AUC: Areas under the curve
CT: Computed tomography
ECV: Extracellular volume
EP: Equilibrium phase
FLR: Future liver remnant
HCC: Hepatocellular carcinoma
ISGLS: International Study Group of Liver Surgery
MELD: Model for end-stage liver disease
mFLR: Measured future liver remnant
PHLF: Posthepatectomy liver failure
ROC: Receiver operator characteristic
ROI: Region of interest
Tbil: Total bilirubin
TLV: Liver volume
TV: Tumor volume
