# Supplementary material for: CT-derived extracellular volume and liver volumetry can predict posthepatectomy liver failure in hepatocellular carcinoma
Source: Insights Imaging. 2023 Sep 12;14:145. doi: 10.1186/s13244-023-01496-5 (PMC10495294; doi:10.1186/s13244-023-01496-5)
Supplement: Supplementary file 1 — Additional file 1: Fig. S1. Sample for CT liver volumetry analysis. A 59-year-old man with HCC, underwent a major hepatectomy (resection of left lobe of liver). In the preoperative baseline axial portal phase CT image(A), handcrafted ROIs were drawn along the margins of the tumor (TV, ROI contoured in orange), the total liver (TLV, ROI contoured in green), and the future liver remnant (FLR volume, ROI contoured in yellow). Volume-rendered image of TV (B) and FLR volume (C) in green, TLV in red, hepatic veins and portal veins in golden. CT Computed tomography, ROI Region of interest, TV Tumor volume, TLV Total liver volume, FLR Future liver remnant. Fig. S2. Boxplot showed a strong correlation between CT-derived ECV and the postoperative pathological fibrosis stage of the background liver (p < 0.001, r = 0.701). CT Computed tomography, ECV Extracellular volume. Fig. S3. Boxplot showed that there was a significant difference in the mean value of CT-derived ECV between subgroup S0-2 and S3-4 (25.34 ± 3.03 vs. 31.17 ± 4.40, p < 0.001). CT Computed tomography, ECV Extracellular volume. Table S1. Intraobserver and interobserver reliability of CT-derived ECV and CT liver volumetry. Abbreviations: CI Confidence interval, CT Computed tomography, ECV Extracellular volume, TLV Total liver volume, TV Tumor volume, FLR Future liver remnant. [file 13244_2023_1496_MOESM1_ESM.docx]

Additional file 1

**
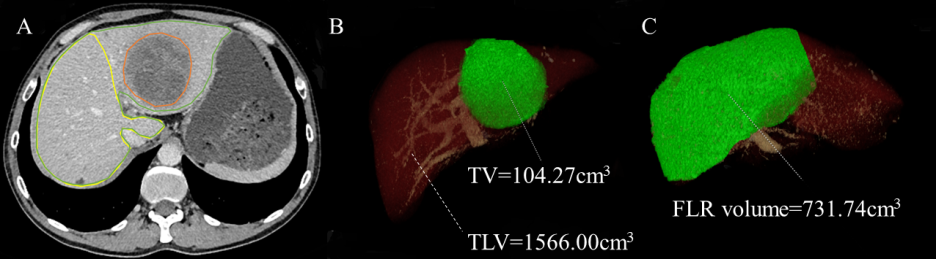
**

**Figure S1.** Sample for CT liver volumetry analysis. A 59-year-old man with HCC, underwent a major hepatectomy (resection of left lobe of liver). In the preoperative baseline axial portal phase CT image (A), handcrafted ROIs were drawn along the margins of the tumor (TV, ROI contoured in orange), the total liver (TLV, ROI contoured in green), and the future liver remnant (FLR volume, ROI contoured in yellow). Volume-rendered image of TV (B) and FLR volume (C) in green, TLV in red, hepatic veins and portal veins in golden. CT, computed tomography; ROI, region of interest; TV, tumor volume; TLV, total liver volume; FLR, future liver remnant.


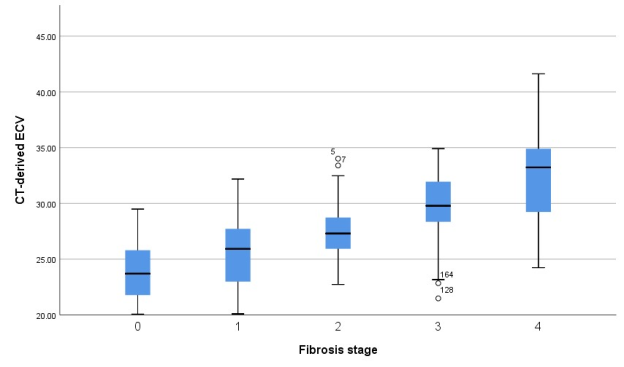


**Figure S2.** Boxplot showed a strong correlation between CT-derived ECV and the postoperative pathological fibrosis stage of the background liver (*P* < 0.001, r = 0.701). CT, computed tomography; ECV, extracellular volume.


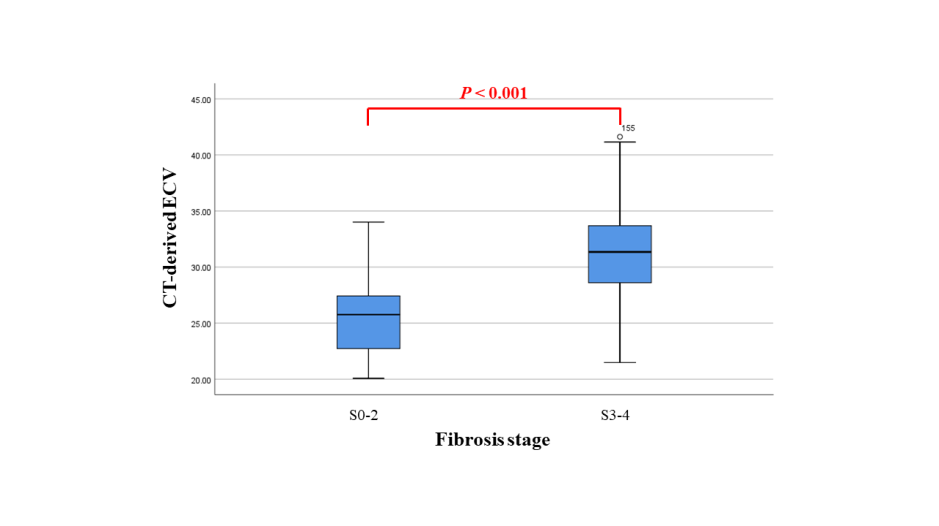


**Figure S3.** Boxplot showed that there was a significant difference in the mean value of CT-derived ECV between subgroup S0-2 and S3-4 (25.34 ± 3.03 vs. 31.17 ± 4.40, *P* < 0.001). CT, computed tomography; ECV, extracellular volume.

**Table S1** Intraobserver and interobserver reliability of CT-derived ECV and CT liver volumetry

| **Variable** | **Training cohort**  **(n = 181)** | | **Internal validation cohort**  **(n = 100)** | | **External validation cohort**  **(n = 112)** | |
| --- | --- | --- | --- | --- | --- | --- |
|  | Intraobserver agreement (95% CI) | Interobserver agreement (95% CI) | Intraobserver agreement (95% CI) | Interobserver agreement (95% CI) | Intraobserver agreement (95% CI) | Interobserver agreement (95% CI) |
| **CT-derived ECV** | 0.989 (0.985, 0.991) | 0.959 (0.946, 0.969) | 0.991 (0.987, 0.994) | 0.974 (0.961, 0.982) | 0.983 (0.976, 0.989) | 0.943 0.919, 0.961) |
| **TV (cm^3^)** | 0.995 (0.993, 0.996) | 0.992 (0.989, 0.994) | 0.992 (0.988, 0.995) | 0.986 (0.979, 0.991) | 0.990 (0.986, 0.993) | 0.977 (0.967, 0.984) |
| **TLV (cm^3^)** | 0.988 (0.984, 0.991) | 0.969 (0.958, 0.977) | 0.983 (0.975, 0.989) | 0.968 (0.952, 0.978) | 0.971 (0.958, 0.980) | 0.964 (0.948, 0.975) |
| **FLR volume (cm^3^)** | 0.987 (0.982, 0.990) | 0.973 (0.964, 0.980) | 0.974 (0.962, 0.982) | 0.966 (0.950, 0.977) | 0.959 (0.941, 0.971) | 0.949 (0.948, 0.965) |

Abbreviations: CI, confidence interval; CT, computed tomography; ECV, extracellular volume; TLV, total liver volume; TV, tumor volume; FLR, future liver remnant.
